# GPs’ views on emergency care treatment plans: an online survey

**DOI:** 10.3399/BJGPO.2023.0192

**Published:** 2024-05-01

**Authors:** Martin Underwood, Angela Noufaily, Hazel Blanchard, Jeremy Dale, Jenny Harlock, Paramjit Gill, Frances Griffiths, Rachel Spencer, Anne-Marie Slowther

**Affiliations:** 1 Warwick Medical School, University of Warwick, Coventry, UK; 2 University Hospitals of Coventry and Warwickshire, Coventry, UK; 3 Forrest Medical Centre, Coventry, UK

**Keywords:** ethics, emergency care and treatment plans, survey and questionnaires, general practice

## Abstract

**Background:**

A holistic approach to emergency care treatment planning is needed to ensure that patients’ preferences are considered should their clinical condition deteriorate. To address this, emergency care and treatment plans (ECTPs) have been introduced. Little is known about their use in general practice.

**Aim:**

To find out GPs’ experiences of, and views on, using ECTPs.

**Design & setting:**

Online survey of GPs practising in England.

**Method:**

A total of 841 GPs were surveyed using the monthly online survey provided by medeConnect, a market research company.

**Results:**

Forty-one per cent of responders' practices used Recommended Summary Plan for Emergency Care and Treatment (ReSPECT) plans for ECTP, 8% used other ECTPs, and 51% used Do Not Attempt Cardiopulmonary Resuscitation (DNACPR) forms. GPs were the predominant professional group completing ECTPs in the community. There was broad support for a wider range of community-based health and social care professionals being able to complete ECTPs. There was no system for reviewing ECTPs in 20% of responders’ practices. When compared with using a DNACPR form, GPs using a ReSPECT form for ECTP were more comfortable having conversations about emergency care treatment with patients (odds ratio [OR] = 1.72, 95% confidence interval [CI] = 1.1 to 2.69) and family members (OR =1.85, 95% CI = 1.19 to 2.87).

**Conclusion:**

The potential benefits and challenges of widening the pool of health and social care professionals initiating and/or completing the ECTP process needs consideration. ReSPECT plans appear to make GPs more comfortable with ECTP discussions, supporting their implementation. Practice-based systems for reviewing ECTP decisions should be strengthened.

## How this fits

Recommended Summary Plan for Emergency Care and Treatment (ReSPECT) is a particular model of emergency care and treatment plan (ECTP), which is currently being implemented across primary and secondary care in many areas of the UK. Little is known about the use of ECTPs in primary care. This research found GPs felt more comfortable having a ReSPECT conversation than other forms of ECTP conversation. Consideration should be given to the potential benefits and challenges of widening the pool of health and social care professionals initiating and/or completing ECTPs, and to strengthening practice-based processes for their review.

## Introduction

Using Do Not Attempt Cardiopulmonary Resuscitation (DNACPR) decisions to help future decision making for people with a life-threatening condition is well established in both primary and secondary care.^1^ These do not, however, convey substantial clinical information, or what an individual’s treatment preferences might be, nor consider which other treatments might, or might not, be appropriate should their clinical condition deteriorate.^2–4^ In response, there has been a move to a more holistic approach to recording recommendations about future treatment decisions with the development of emergency care treatment plans (ECTPs). These plans encompass broader clinical decision making, while still describing DNACPR recommendations. Several models of ECTP have been developed by individual NHS trusts or regional healthcare systems in the UK.^5–8^ In 2016, the Resuscitation Council UK (RCUK) developed a model of ECTP that was intended to be used nationally across primary and secondary care.^9,10^ By 2023, this model, the Recommended Summary Plan for Emergency Care and Treatment (ReSPECT), had been adopted to some extent in 65% of integrated care systems in England (personal communication, RCUK).

An evaluation of ReSPECT in early adopting acute NHS trusts identified challenges, with clinicians suggesting that the conversation, and completion of a ReSPECT plan, would be better in primary care with conversation(s) taking place over a period of time, with a GP with whom the patient has an ongoing relationship.^11^ Focus groups with GPs identified challenges to using ReSPECT in primary care. GPs with experience of completing ReSPECT forms conceptualised them as end-of-life planning documents, limiting the population for whom a plan might be initiated. Recommendations on GP-initiated plans differed from those completed in hospitals, reflecting the context in which they were expected to be used.^12^

The COVID-19 pandemic increased focus on the role of ECTP. Regulatory authorities identified the importance of individualised conversations with patients about future treatment decisions, carried out by healthcare professionals with the requisite skills, knowledge, and confidence.^13,14^

Little is known about how GPs view and make use of ECTPs with their patients. We report a national survey measuring GPs’ use of ECTPs, their views on using ECTPs in primary care; their readiness to complete plans with their patients, patients' families, or someone important to the patient (henceforth included within the term 'families'); and the factors that might influence this process.

## Method

The survey is part of a larger mixed-methods evaluation of the use of ECTPs in primary care.^15^ Informed by our qualitative work in GP practices, and with involvement from our patient and public advisory group, we developed a questionnaire survey to measure the views of GPs working in England regarding the use of ECTPs in primary care (Table 1). For the survey, we included DNACPR forms as a type of ECTP, albeit one limited to a single emergency treatment decision.

**Table 1.** Responder characteristics, totals, and percentages

Characteristic	Total (*N* = 841)		National data
**Age, years**			
≤35	39	5%	11%^a^
36–45	319	38%	36%
46–55	318	38%	30%
≥56	165	20%	22%
**Gender**			
Male	446	53%	42%^b^
Female	385	46%	57%
Other	1	0%	1%
Prefer not to say	9	1%	–
**Current role**			
GP partner or principal	419	50%	42%^c^
Salaried GP	255	30%	27%
Locum GP	156	19%	–
GP registrar	11	1%	–
**English NHS region**			
London	114	14%	16%^d^
South West	85	10%	11%
South East	142	17%	15%
West Midlands	92	11%	19%
East Midlands	62	7%	
East of England	93	11%	11%
Yorkshire and Humber	94	11%	15%
North East	44	5%	
North West	115	14%	13%
**Type of area**			
Major conurbation	157	19%	39%^e^
Large town or city	124	15%	
Medium town or city	207	25%	52%
Small town or city	254	30%	
Hamlet	94	11%	8%
Other	5	1%	–
**Practice size**			
≤5000	89	11%	8%^f^
5001–7500	129	15%	14%
7501–10 000	181	22%	18%
10 001–12 500	147	17%	17%
≥12 501	295	35%	44%
**Time since completing GP training**			
0–5 years	51	6%	–^g^
6–10 years	140	17%	–
11–15 years	225	27%	–
16–20 years	160	19%	–
>20 years	265	32%	–

^a^Data from https://digital.nhs.uk/data-and-information/publications/statistical/general-and-personal-medical-services, age bands not exact matches, 1% unknown, excludes trainees. ^b^Data from https://digital.nhs.uk/data-and-information/publications/statistical/general-and-personal-medical-services, other includes unknown, excludes trainees and locums. ^c^Data from https://digital.nhs.uk/data-and-information/publications/statistical/general-and-personal-medical-services, denominator all GPs, locums and trainees not reported because of differences in defintions. ^d^Population distribution as proxy for GP practice location from https://assets.publishing.service.gov.uk/government/uploads/system/uploads/attachment_data/file/1028819/Rural_population__Oct_2021.pdf. ^e^Data from https://digital.nhs.uk/data-and-information/publications/statistical/patients-registered-at-a-gp-practice/february-2023. ^f^Data from https://digital.nhs.uk/data-and-information/publications/statistical/general-and-personal-medical-services, other includes unknown, excludes trainees and locums. ^g^No suitable data source identifed.

Key questions of interest were to identify which factors might predict how comfortable GPs were in having ECTP conversations with a patient or family member, assessed using 5-point Likert scales. After developing our initial questions, we refined these using think-aloud interviews with six GPs.

We outsourced data collection to medeConnect, a market research company providing a monthly online survey of 1000, regionally representative UK GPs (Appendix 1).^16^ There were no restrictions on multiple GPs from the same practice completing the survey. The final questions, formatted in an online survey, were tested by the company and the research team. These are presented in Tables 2 3 4.

**Table 2.** Totals and percentages for the emergency care and treatment planning form completion (*N* = 841)

		*n*	% (95% CI)
**What form of emergency care and treatment plans does your practice use?**			
ReSPECT		345	41 (38 to 44)
DNACPR		426	51 (47 to 54)
Other		70	8 (6 to10)
**Who completes emergency care and treatment plans within your practice?**			
GP		780	93 (91 to.94)
GP trainee		0	–
Practice nurse		79	9 (7 to11)
Advanced nurse practitioner		234	28 (25 to 31)
Specialist nurse practitioner for elderly care		140	17 (14 to 19)
**Who do you think should be able to complete emergency care treatment plans in a GP practice?**			
GP		797	95 (93 to 96)
GP trainee		522	62 (59 to 65)
Practice nurse		350	42 (38 to 45)
Advanced nurse practitioner		648	77 (74 to 80)
Specialist nurse practitioner for elderly care		663	79 (76 to 82)
Emergency care practitioner		550	65 (62 to 69)
**Who do you think should be able to complete emergency care treatment plans in the community?**			
Specialist nurse practitioner for palliative care		802	95 (94 to 97)
Other specialist nurse practitioner		691	82 (80 to 85)
Community matron or senior nurse practitioner for community care		690	82 (79 to 85)
District nurse		467	56 (52 to 59)
Senior care home staff		207	25 (22 to 28)
Senior nurses in nursing home		430	51 (48 to 55)
**When would you consider completing an emergency care and treatment plan for a patient?**			
When a patient reaches a certain age		199	24 (21 to 27)
When a patient is diagnosed with a life-threatening condition		722	86 (83 to 88)
When a patient is diagnosed with a chronic long-term condition		509	61 (57 to 64)
When a patient is severely disabled		497	59 (56 to 62)
When you think a patient is likely to die within 12 months		813	97 (95 to 98)
When a patient is admitted to a care home		596	71 (68 to 74)
**When do you review an emergency care and treatment plan for a patient?**			
When a patient requests it		477	57 (53 to 60)
When a patient is discharged from hospital with an ECTP		389	46 (43 to 50)
Annually		309	37 (33 to 40)
6-monthly		104	12 (10 to 15)
Annually, or 6 monthly, or ≥75 health check		486	58 (54 to 61)
During or following the annual health check for patients aged ≥75 years		238	28 (25 to 31)
When you think the patient’s health has changed		595	71 (68 to 74)
My practice does not have a system for reviewing ECTP forms		169	20 (17 to 23)

DNACPR = Do Not Attempt Cardiopulmonary Resuscitation. ECTP = emergency care treatment planning. ReSPECT = Recommended Summary Plan for Emergency Care and Treatment.

**Table 3.** Attitudes to emergency care treatment planning (*N* = 841)

	Strongly agree	Agree	Neither agree nor disagree	Disagree	Strongly agree
*Having a plan means that the patient might not get a treatment that could save their life*					
	14	128	131	375	193
	2%	15%	16%	45%	23%
*Having a plan can avoid the patient’s family having to make difficult decisions for them*					
	200	489	104	38	10
	24%	58%	12%	5%	1%
*There is a serious risk that the plan could be out of date when implemented and not reflect the patient’s current views*					
	33	315	277	200	16
	4%	37%	33%	24%	2%
*The patient’s current health condition may not be reflected in the plan when implemented and there is a serious risk it could be out of date*					
	34	391	235	166	15
	4%	46%	28%	20%	2%
*Having a plan ensures that treating clinicians know the patient’s wishes*					
	224	527	80	9	1
	27%	63%	10%	1%	0%

**Table 4.** How comfortable or uncomfortable do you feel having conversations about an emergency care and treatment plans? (*N* = 841)

	Very comfortable	Fairly comfortable	Neither comfortable nor uncomfortable	Fairly uncomfortable	Very uncomfortable
*How comfortable or uncomfortable do you feel having conversations about an emergency care and treatment plan with* *patients* *?*					
	251	428	112	46	4
	30%	51%	13%	5%	0%
*How comfortable or uncomfortable do you feel having conversations about an emergency care and treatment plan with the* *patient’s family* *(or someone important to the patient)?*					
	227	441	122	45	6
	27%	52%	15%	5%	1%

### Sample size and statistical analysis

For a binary outcome (very comfortable or fairly comfortable versus all other responses) a sample size of 1000 (the size of the medeConnect monthly survey) would: if 50% were ‘comfortable’, provide precision of 6.2%; or, if 80% were comfortable, provide a precision of 5%.

In addition to descriptive statistics for each question, we present logistic regression analyses investigating the variables associated with how comfortable GPs are having ECTP discussions with patients or their families. We initially did unadjusted logistic regression analyses with gender, GP role, NHS region, type of area (that is, major conurbation, large town), years since completion of GP training, and use of ReSPECT form versus DNACPR or another ECTP as explanatory variables. We then constructed a fully adjusted logistic regression model including all the explanatory variables. As a sensitivity analysis, we repeated this using a backward elimination approach.

## Results

The survey ran in November 2022. Only the 841 (of 1000) GPs surveyed who practised in England were invited to complete it (Appendix 2). We did not achieve our original sample because of the need to gain additional ethics approval to include the devolved nations. Responders’ demographic characteristics were broadly representative of GPs in England, although males and GP partners or principals were overrepresented (Table 1). Just over half (51%) of responders reported their practice used standalone DNACPR forms. ReSPECT forms were used by 41%, and 8% used other ECTP forms (Table 2). There were substantial regional differences in the forms used ranging from just over three-quarters of GPs in East (79%) and West Midlands (80%) using ReSPECT forms to DNACPR forms being predominantly used by GPs in London (76%), the North East (75%), and North West (77%) (Supplementary Table S1).

Overwhelmingly (93%), responders reported that GPs completed ECTPs within their practices, and they felt that GPs should be able to complete them (Table 2). However, there were substantial disparities between who was reported as completing ECTPs and who they thought could complete them. Consistently, responders suggested that a wider range of healthcare professionals should be able to complete the forms. For example, no responders reported GP trainees (registrars) completing ECTPs in their practice, whereas 62% felt they should be able to do this task (Table 2). Just over three-quarters (77%) of responders thought that advanced nurse practitioners should be able to complete ECTPs but only about one-quarter (28%) reported that this currently happened (Table 2). Similarly, there was broad support for a wide range of community-based health and social care professionals being able to complete ECTPs; for example, more than 80% supported senior nurses completing them (82%–95%), half supported less senior nurses completing the forms (51%–56%), and one-quarter (25%) supported senior care home staff to do this task (Table 2).

When GPs would consider completing an ECTP was primarily influenced by the patient’s health state; 97% would consider completing a form if they felt the patient had a life expectancy of less than 1 year, 86% when a patient has been diagnosed with a life-threatening condition, and 71% when a patient entered a care home. Just under one in four (24%) GPs would consider completing a plan based on the patient’s age alone. An ECTP was considered by fewer responders for people who were severely disabled (59%) or living with a long-term condition (61%) (Table 2).

A mixed pattern was seen for when GPs might consider reviewing ECTPs. Strikingly, one in five (20%) responders reported that their practices had no system for reviewing forms. Only a minority had routine systems in place for reviewing these; annually (37%), 6-monthly (12%), or at the annual health check for patients aged ≥75 (28%). Even when there was a patient request (57%), or a change in health state (71%), it was far from standard practice to review forms. Only 46% would consider reviewing an ECTP following a hospital admission (Table 2).

Overall, ECTP was viewed positively; 89% agreed that having a plan ensures treating clinicians know the patient’s wishes and 82% agreed it can avoid patients’ families making difficult decisions. Nevertheless, half (51%) agreed that a patient’s current health condition may not be reflected in the plan when implemented and there is a serious risk it could be out of date (Table 3).

Considering the last time they had completed an ECTP, a small minority (9%) reported that a family member was not involved. Most commonly this was because the patient had capacity 54/72 (75%), although 18 (25%) reported that the family was not available and 11 (15%) that the patient didn’t want the family involved. One responder reported that the family did not want to be involved (Supplementary Table S2).

GPs reported being at least fairly comfortable having ECTP conversations with both the patient (81%) and the patient’s family (79%) (Table 4).

In our adjusted logistic regression analyses for conversations with patients, locum and salaried GPs were substantially (around 48%) less likely to be comfortable having ECTP conversations compared with partner or principal GPs; odds ratio (OR) = 0.51 (95% confidence interval [CI] = 0.31 to 0.82) and 0.53 (95% CI = 0.34 to 0.82), respectively (Table 5, Supplementary Table S3). For conversations with family members the difference was only statistically significant for salaried GPs; OR 0.58 (95% CI = 0.38 to 0.88; Supplementary Table S4).

**Table 5.** Odds ratios (95% confidence intervals [CIs]) and *P* values of the predictors for being comfortable having emergency care and treatment planning conversations with patients (*n* = 841)

		Comfortable (*n*)	Adjusted analysis	
			**Odds ratio (95% CI)**	***P*** **value**
**Gender**				
Female		81% (385)	1	
Male		81% (446)	0.85 (0.59 to 1.24)	0.407
Other		100% (1)	–	0.995
Prefer not to say		67% (9)	0.36 (0.08 to 1.53)	0.166
**Current role**				
GP partner or principal		85% (419)	1	
GP registrar		100% (11)	–	0.984
Locum GP		73% (156)	0.51 (0.31 to 0.82)	0.006
Salaried GP		77% (255)	0.53 (0.34 to 0.82)	0.004
**NHS region**				
London		69% (114)	1	
East of England		77% (93)	1.24 (0.49 to 3.15)	0.655
West Midlands		78% (92)	1.29 (0.53 to 3.16)	0.577
North West		77% (115)	1.54 (0.68 to 3.49)	0.297
Yorkshire and Humber		83% (94)	1.80 (0.70 to 4.63)	0.222
South East		83% (142)	2.08 (0.84 to 5.17)	0.115
East Midlands		89% (62)	2.47 (0.78 to 7.86)	0.124
North East		89% (44)	4.10 (1.22 to 13.8)	0.023
South West		91% (85)	4.30 (1.45 to 12.7)	0.008
**Type of area**				
Large town or city		84% (124)	1	
Major conurbation		74% (157)	1.01 (0.43 to 2.35)	0.987
Medium town or city		80% (207)	0.75 (0.40 to 1.40)	0.361
Small town or city		83% (254)	0.76 (0.41 to 1.41)	0.391
Village or hamlet		85% (94)	1.16 (0.53 to 2.54)	0.714
Other		60% (5)	0.30 (0.04 to 2.22)	0.237
**Practice size**				
≤5000		75% (89)	1	0
5001–7500		78% (129)	0.98 (0.49 to 1.96)	0.961
7501–10 000		76% (181)	0.85 (0.44 to 1.62)	0.614
10 001–12 500		83% (147)	1.40 (0.68 to 2.86)	0.360
≥12 501		85% (295)	1.60 (0.83 to 3.11)	0.161
**TIme since completion of GP training**				
0–5 years		90% (51)	1	
6–10 years		83% (140)	0.62 (0.21 to 1.82)	0.388
11–15 years		81% (225)	0.54 (0.19 to 1.52)	0.246
16–20 years		80% (160)	0.46 (0.16 to 1.35)	0.158
>20 years		78% (265)	0.43 (0.15 to 1.19)	0.105
**Emergency care and treatment form used**				
DNACPR		77% (426)	1	
ReSPECT		86% (345)	1.72 (1.10 to 2.69)	0.017
Other		80% (70)	1.00 (0.51 to 1.95)	0.998

When compared with London, GPs in the South West and the North East were substantially more likely (odds around 4.2 times greater) to be comfortable with ECTP conversations; OR = 4.30 (95% CI = 1.45 to 12.7) and 4.10 (95% CI = 1.22 to 13.8), respectively. For conversations with family members, GPs from the South East, East Midlands, or Yorkshire and Humber were also more comfortable than GPs from London (Table 5, Supplementary Table S4).

GPs using a ReSPECT form were more comfortable having these conversations with patients (72% more odds) and family members (85% more odds) when compared with GPs using a DNACPR form; OR = 1.72 (95% CI = 1.10 to 2.69) and OR = 1.85 (95% CI = 1.19 to 2.87), respectively. Results from our sensitivity analysis using a backwards elimination model were not materially different (Supplementary Tables S5 and S6).

## Discussion

### Summary

This study shows that ECTPs have become a standard part of general practice with 100% of responders reporting using some form of ECTP. Nevertheless, the finding that just over half (51%) of our responders are still using standalone DNACPR forms is potentially a cause for concern, when the limitations of DNACPR for making holistic patient-centred decisions have been recognised since at least 2016.^17^

GPs who used ReSPECT when compared with DNACPR were more likely to feel comfortable in having ECTP conversations with patients and their relatives. The main trigger for initiating an ECTP conversation is diagnosis of a life-limiting or life-threatening condition. While completion of an ECTP in primary care is currently carried out predominantly by GPs, responders suggested that this could be carried out by a much broader range of health and social care professionals.

Responders were very supportive of a wider spectrum of health and social care professionals being able to complete ECTPs. Support for specialist nurse practitioners for palliative care completing these forms is not surprising. The finding that a substantial minority (25%) of GPs support senior care home staff completing ECTP forms is perhaps more surprising, as these are not designed for completion by non-clinicians. Possibly our responders had in mind senior care home staff having the initial conversations with their residents rather than formal completion of the form without clinician input. Indeed, an interview study of GPs and care home staff found that GPs value the input of care home staff in ReSPECT conversations.^18^ While care home staff were generally positive about being involved, they had concerns about taking responsibility for the form’s content.^18^

Most responders reported the patient’s clinical condition as the stimulus for initiating ECTP conversations, predominantly in the context of life-limiting diagnosis or terminal prognosis. This conceptualises ECTPs as being associated with end-of-life care. This contrasts with how its developers envisaged ReSPECT but is consistent with the previous studies of the ReSPECT process.^12,19,20^ It is unclear whether time constrains the GP staff to focus on patients who are perceived to be likely to have an acute need for emergency care in the foreseeable future, or they are conflating ECTPs with advance care planning. How GPs conceptualise ECTPs may affect their views on who can complete them and how often they need reviewing. We are exploring this question in our related qualitative study.^15^

The finding that one in five (20%) practices have no system for reviewing ECTPs with only 58% having any routine system for review is of concern. This is concerning particularly since 41% of our responders agreed that there might be a serious risk of the plan being out of date and not reflecting the patient’s views, and half (50%) felt the patient’s current health condition might not be reflected in the plan when implemented.

Caution is needed when interpreting the apparent regional differences observed in how comfortable GPs felt in having ECTP conversations because of the large number of comparisons and small numbers in some groups. Nevertheless, there appears to be differences between London, and both the South West and the North East. This might reflect the impact of the presence or absence of local ECTP initiatives. For example, few London GPs use ReSPECT forms and will not have been exposed to ReSPECT training while in the North East there has been a long-standing regional integrated approach to making care decisions in advance that includes emergency care treatment planning, with an associated education initiative (Supplementary Table S1).^5^

The finding that our data show that GPs using the ReSPECT forms are more comfortable with ECTP conversations is important. What we do not know from this study is why they felt more comfortable and whether this increased comfort reflects the structure of the form itself, the added value of any training related to its implementation, or whether early adopters were already more comfortable. We do not know if this translates into better quality decisions or improved patient outcomes.

### Strengths and limitations

We obtained a high quality dataset with no missing data. Our responders were representative of England in terms of region, age, practice size, years since qualification, and region (Table 1). Nevertheless, outsourcing data collection to a market research company working through a commercially funded, free-to-use website may have introduced bias into the sample selection. GPs signed up to the online survey with the Doctors.net.uk website may not be representative of all GPs in terms of their commitment to continuing professional development (CPD) and up-to-date practice. We do not know if we have had responses from multiple GPs working in the same practice. Females and non-principals were underrepresented in this survey. This needs to be set against the known challenges of sending ‘cold’ surveys to GPs in terms of response rate and data quality (Appendix 2). Some caution is needed interpreting regression analyses because of the large number of comparisons made. Given that many GPs are using DNACPR forms rather than ReSPECT (or other ECTP) forms, it is possible some reflect their experiences of DNACPR decision making rather than emergency care and treatment planning. Overall, our approach has delivered a robust overview of GPs’ views on this difficult topic. Nevertheless, we have no data on what actually happens in general practice.

### Comparison with existing literature

This is the first survey of GPs’ use of ECTP.

### Implications for practice

ECTPs are seen as providing benefit to patients by GPs. Using ReSPECT makes GPs more comfortable with ECTP discussions. Nevertheless, just over half of our responders still use DNACPR forms. Future implementation of ECTP in primary care should consider its conceptualisation and use in relation to advance care planning more generally to ensure people who may benefit are not excluded from conversations. Patients and their informal carers prefer healthcare professionals to initiate an advance care planning conversation, and their views on initiation and completion of ECTPs may be similar.^21^ Given our findings, widening the pool of health and social care professionals involved in ECTP conversations should be considered; however, further work is needed to explore the acceptability of this approach to ECTP discussions for patients, their families, and the professionals involved.

Systems for reviewing prior recommendations need to be strengthened.

## Data

All requests for data should be sent to the Warwick Clinical Trials Unit data access team (wctudataaccess@warwick.ac.uk). Access to anonymised data may be granted following review.
